# Smart Portable Device Based on the Utilization of a 2D Disposable Paper Stochastic Sensor for Fast Ultrasensitive Screening of Food Samples for Bisphenols

**DOI:** 10.3390/s23010314

**Published:** 2022-12-28

**Authors:** Raluca-Ioana Stefan-van Staden, Irina-Alina Chera-Anghel, Damaris-Cristina Gheorghe, Jacobus (Koos) Frederick van Staden, Marius Badulescu

**Affiliations:** 1 Laboratory of Electrochemistry and PATLAB, National Institute of Research for Electrochemistry and Condensed Matter, 202 Splaiul Independentei Str., 060021 Bucharest, Romania; 2 Faculty of Chemical Engineering and Biotechnologies, University Politehnica of Bucharest, 060042 Bucharest, Romania; 3 Low Temperature Plasma Laboratory, National Institute for Lasers, Plasma and Radiation Physics (NILPRP), 409 Atomistilor St., 077125 Magurele, Romania

**Keywords:** bisphenol, food, 2D disposable stochastic sensor, portable device

## Abstract

Since the determination of the high toxicity of bisphenol A, alternative structures for bisphenols have been synthesized, resulting in bisphenols C, E, F, S, and Z. These bisphenols have replaced bisphenol A in plastic bottles, toys, and cans used for preserving food. Later, the toxicity and negative effects of all of these bisphenols on people’s health were proven. Therefore, there is a need for a fast ultrasensitive screening method that is able to detect the presence of these bisphenols in any condition directly from food samples. This paper presented a disposable device based on the utilization of a 2D disposable paper stochastic sensor for the fast ultrasensitive screening of food samples for bisphenols A, C, E, F, S, and Z. The 2D disposable sensor was obtained by the deposition of graphene and silver nanolayers on paper using cold plasma. Furthermore, the active side of the sensor was modified using 2,3,7,8,12,13,17,18-octaethyl-21H,23H Mn porphyrin. The limits of quantification of these bisphenols were 1 fmol L^−1^ for bisphenols C and E, 10 fmol L^−1^ for bisphenols A and F, 10 pmol L^−1^ for bisphenol S, and 1 pmol L^−1^ for bisphenol Z. The recoveries of these bisphenols in milk, canned fruits, vegetables, and fish were higher than 99.00% with RSD (%) values lower than 1.50%.

## 1. Introduction

Characterized as a ‘pseudo-persistent’ chemical, bisphenol A (BPA) has led to its potential spread and accumulation in a variety of environmental matrices. Exposure to BPA has led to widespread effects on human health because it has estrogenic activity, causing reproductive dysfunction, endometrial hyperplasia, recurrent abortions, abnormal karyotypes, and polycystic ovary syndrome. Therefore, different derivatives such as bisphenol C (BPC), bisphenol E (BPE), bisphenol F (BPF), bisphenol S (BPS), and bisphenol Z (BPZ) were synthesized with the understanding that they may be less toxic, and with no effects on human health. Unfortunately, different clinical and toxicological studies have shown that all of these bisphenols have endocrine disrupting effects, cytotoxicity, genotoxicity, reproductive toxicity, dioxin-like effects, and neurotoxicity [1,2,3,4,5,6,7,8]. Furthermore, the half-life-time of these bisphenols varies from a few hundred days to a few thousand days [2]. Because bisphenols can pass into food, especially by heating/preserving the recipients, and from the food into the body, there is a need for their simultaneous determination in food using fast, ultrasensitive, easy-to-use screening methods for their identification and quantification.

To date, numerous methods of analysis have been proposed for the assay of BPA in different samples (water, food, biological samples) [9,10,11,12] as well as for the assay of other bisphenols [13,14,15,16,17,18]. Chromatographic methods of analysis [10,15,18], spectrophotometric methods of analysis [12], and electrochemical methods of analysis [9,11,13,17] have also been proposed for the determination of bisphenols.

The novelty of the work is the design of a screening test based on the simultaneous detection of bisphenols using stochastic sensors as detection tools. A portable device based on the utilization of a 2D disposable paper stochastic sensor for the fast ultrasensitive screening of food samples for BPA, BPC, BPE, BPF, BPS, and BPZ is proposed in this paper. The 2D sensors were designed to be cost-effective: the graphene and silver layers were deposited using cold plasma on copy paper, which can be used for the assay in the same run of all six bisphenols. The role of the C–Ag nanocomposite deposition was conducted by considering that high conductivity (assured by the Ag) is necessary on an established C-based matrix at a nanofilm level. To obtain the stochastic response, 2,3,7,8,12,13,17,18-octaethyl-21H,23H Mn porphyrin was used; this porphyrin forms molecular aggregates [19] that are able to provide the pores needed for the stochastic response.

## 2. Materials and Methods

### 2.1. Materials and Reagents

BPA, BPC, BPE, BPF, BPS, BPZ, and 2,3,7,8,12,13,17,18-octaethyl-21H,23H Mn porphyrin were purchased from Sigma Aldrich. All solutions were prepared in phosphate buffer solution (PBS, pH = 7.50). Bisphenol solutions of 10^−2^ to 10^−18^ mol L^−1^ were prepared using the serial dilution method.

### 2.2. Design of the Disposable Stochastic Sensor

**Scheme 1:**
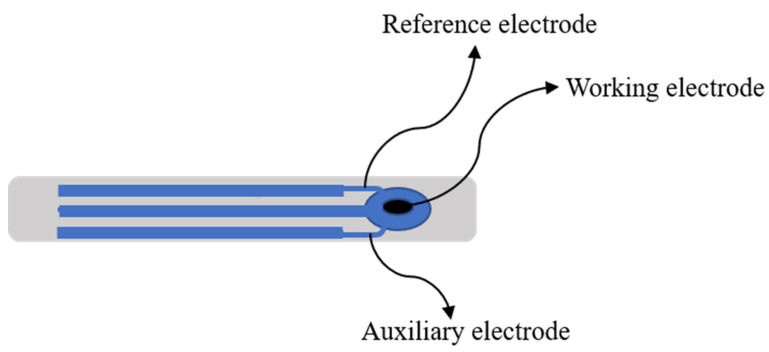
The 2D disposable sensor.

In the present work, carbon–Ag (C–Ag) nanocomposite thin films were deposited on a flexible substrate as part of the disposable stochastic sensor design. C–Ag nanocomposite thin films based on two materials (C = 99.98%, Ag = 99.9%) were prepared on A4 printing paper (commercially copy paper) using a cold plasma method, as described previously [20,21]. The same method was used to deposit a thin layer of Ag/AgCl (as reference electrode) and platinum (as auxiliary electrode) to provide the combined disposable 2D sensor (Scheme 1).

The electrical parameters used for the ignition of a single plasma plume from both C–Ag materials were as follows: 50 A filament current, 1.8 A plasma current, and 400 V plasma voltage. The A4 printing paper substrate was set at a 35 cm distance from the target and the work pressure was 2 × 10^−5^ mBar. During the deposition, the substrate rotated at a speed of 50 rpm at room temperature. The thickness of the films was 8 nm from the cross-sectional scanning electron microscopy (SEM) observation on a Si wafer.

**Figure 1:**
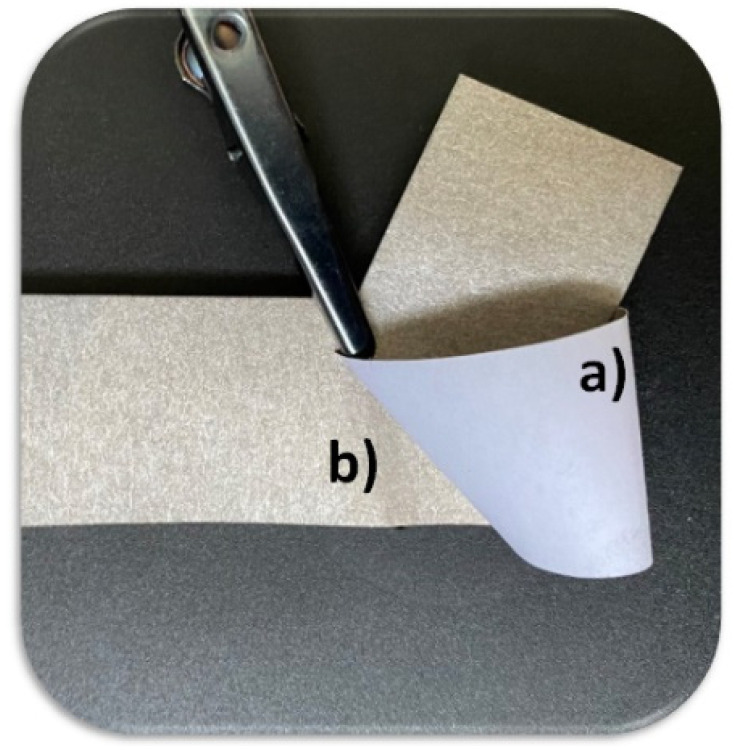
Picture of the curved paper substrate (**a**) uncoated and (**b**) coated with a C–Ag nanocomposite thin film on the paper.

Digital photos of the coated and uncoated C–Ag nanocomposite film on the copy paper substrate are shown in Figure 1.

The flexibility of the copy paper substrate after the coating process with the nanocomposite thin film is represented in Figure 1.

**Figure 2:**
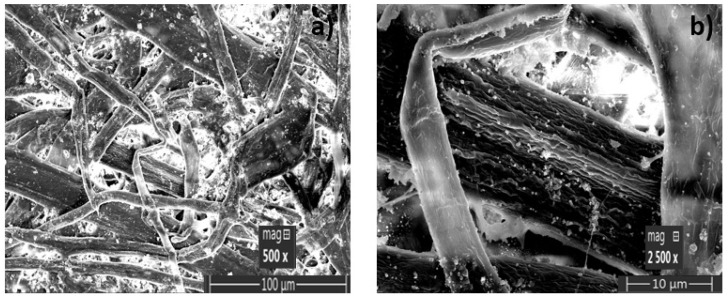
Top-view scanning electron microscope images with different magnification: (**a**) 500×—scale bar 100 µm, and (**b**) 2500×—scale bar 10 µm, for the C–Ag nanocomposite thin films deposited on the copy paper substrates.

The sample was morphologically investigated using SEM at a voltage of 15 kV by using secondary electrons and a pressure of 1 × 10^−3^ Pa. The sample was also investigated for its elements with the help of an EDX detector at a voltage of 10 kV to highlight all the elements that appeared in the sample (the same pressure of 1 × 10^−3^ Pa). SEM images at 2500× and 500× of the C–Ag nanocomposite thin films are shown in Figure 2a,b. The SEM top view image in Figure 2a,b suggests the presence of the C–Ag nanocomposite thin film on the paper fibers and in the apertures between the paper fibers.

**Figure 3:**
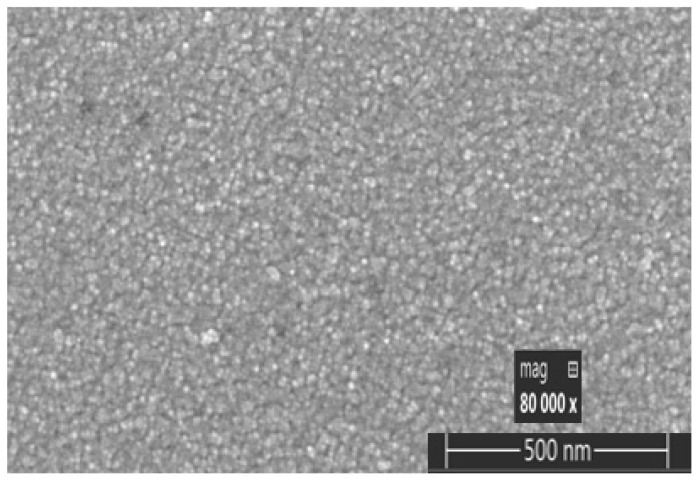
SEM images of the C–Ag nanocomposite thin films deposited on a silicon wafer.

Some nanoparticles seemed to be attached to the paper fibers. SEM analysis of the C–Ag nanocomposite thin film coated onto a silicon wafer fixed on a paper substrate as a reference sample for the film thickness measurements was also conducted and is shown in Figure 3. This sample revealed the formation of well-organized, uniform, and highly dense spherical nanostructures of both materials, carbon and silver, proving that the materials deposited formed a good support for the modifiers (modifiers in the stochastic sensors are those responsible for the sensors’ response, while the support had the role of keeping the channels of the modifier in the best shape).

**Figure 4:**
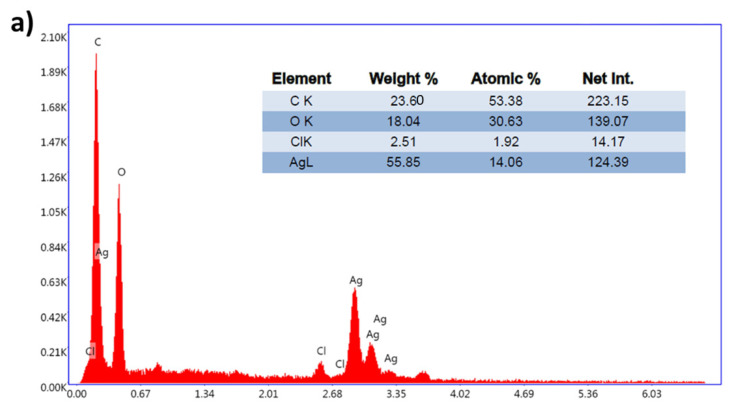

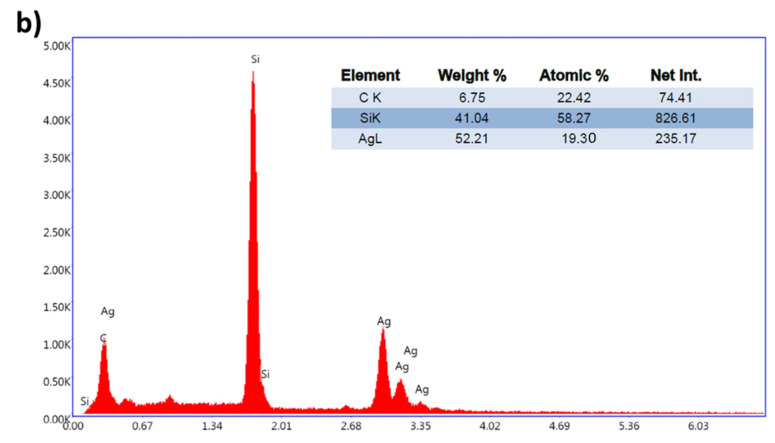
EDAX spectrum and the elemental composition of deposition onto the (**a**) copy paper substrates and (**b**) silicon wafer, respectively.

The EDAX spectra of one of the ten pieces analyzed are presented in Figure 4a,b, which are consistent with the proposed composition. The results of the EDAX elemental microanalysis of the C–Ag onto copy paper and on the silicon wafer are listed in Figure 4a,b, respectively. The results confirm the presence of all elements occurring in the coatings used (C and silver) including other chemical elements such as C, O, and Cl, corresponding to the copy paper substrate. The variation in the percentage of C found in the 10 pieces measured was 1.02%, while the variation in the percentage of Ag in the 10 pieces measured was 1.11%.

To modify the active surface of the working sensor, a drop of 2,3,7,8,12,13,17,18-octaethyl-21H,23H Mn porphyrin solution (10^−3^ mol L^−1^) was added to the active side, and left to dry for 24 h before use.

### 2.3. Stochastic Method

**Figure 5:**
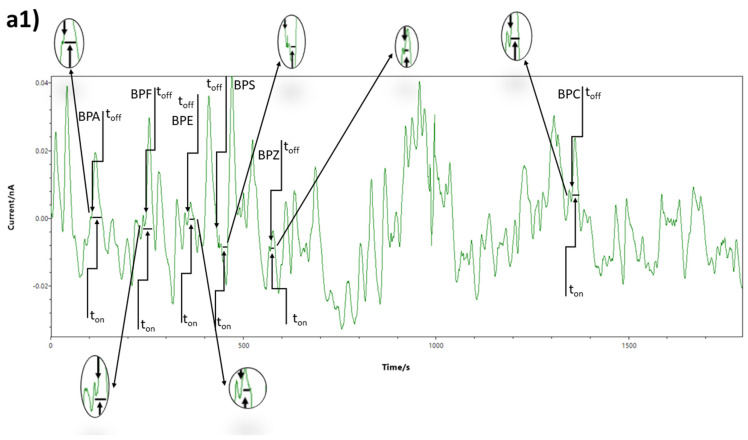

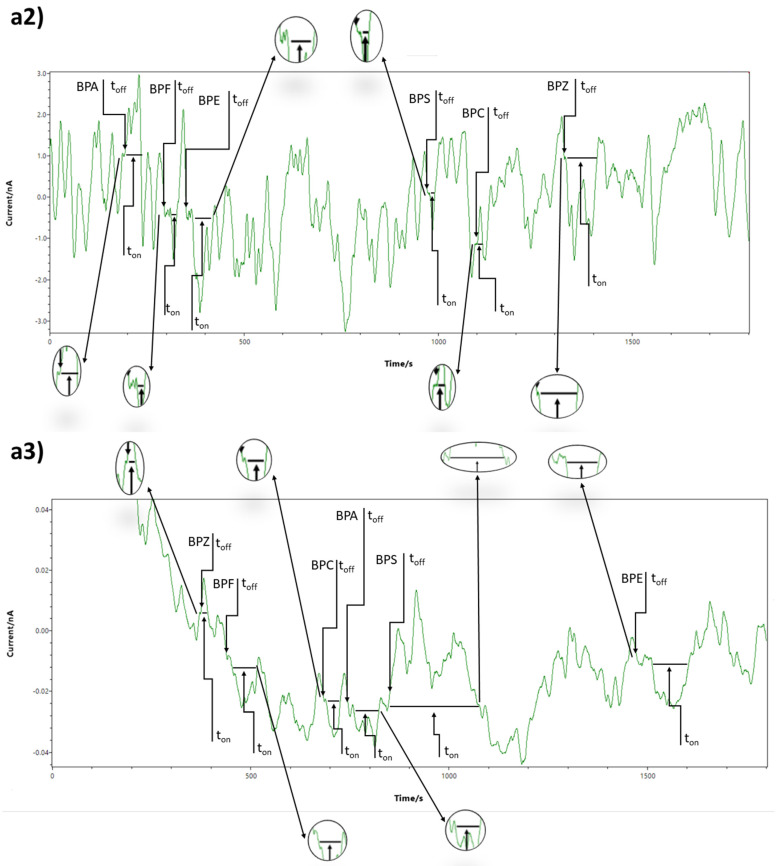

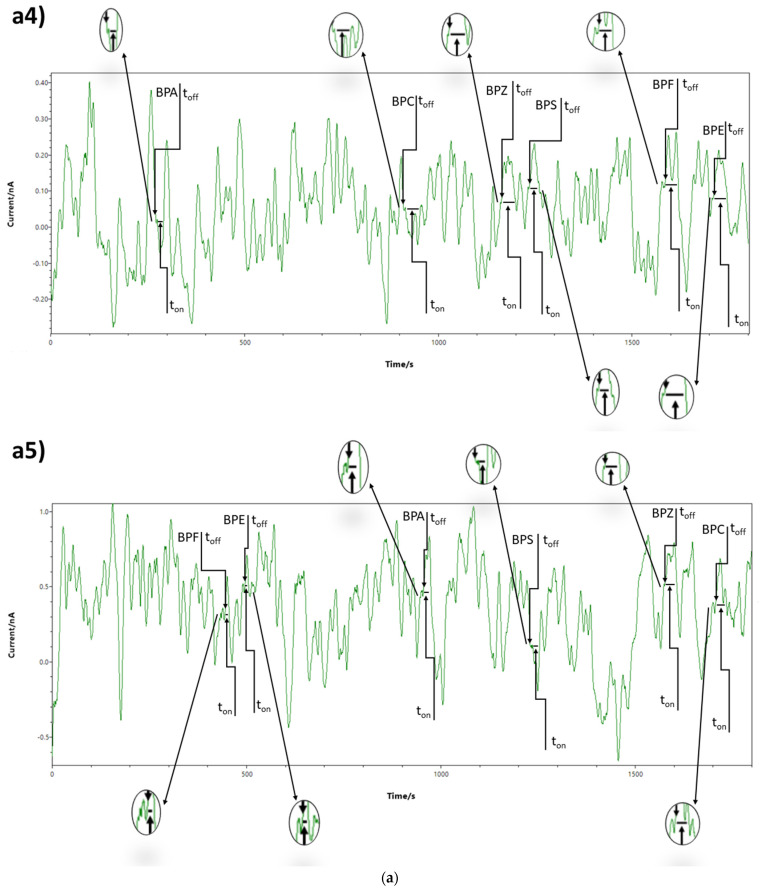

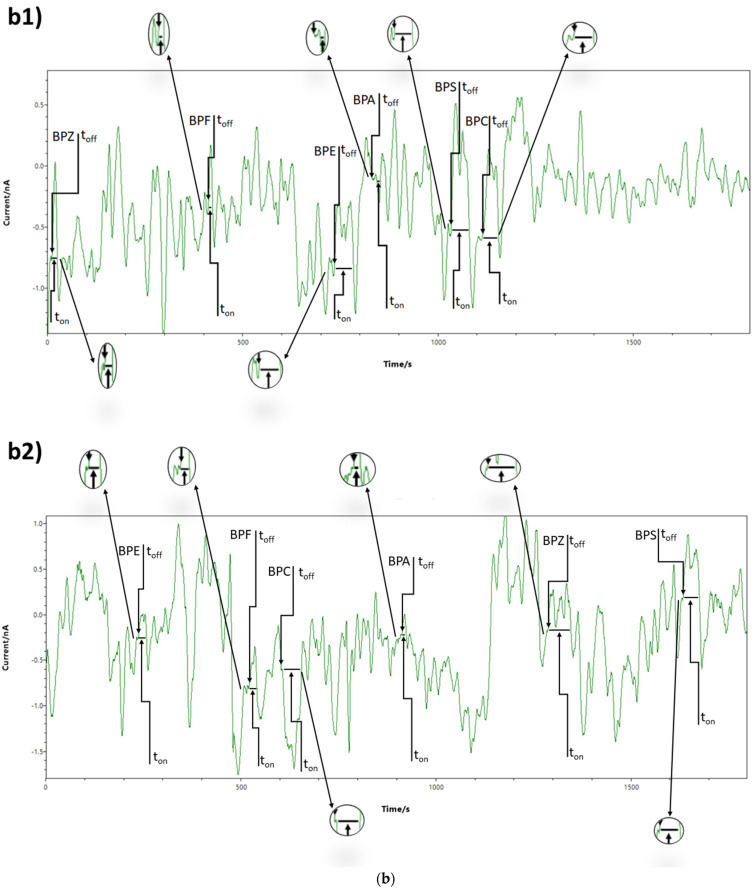

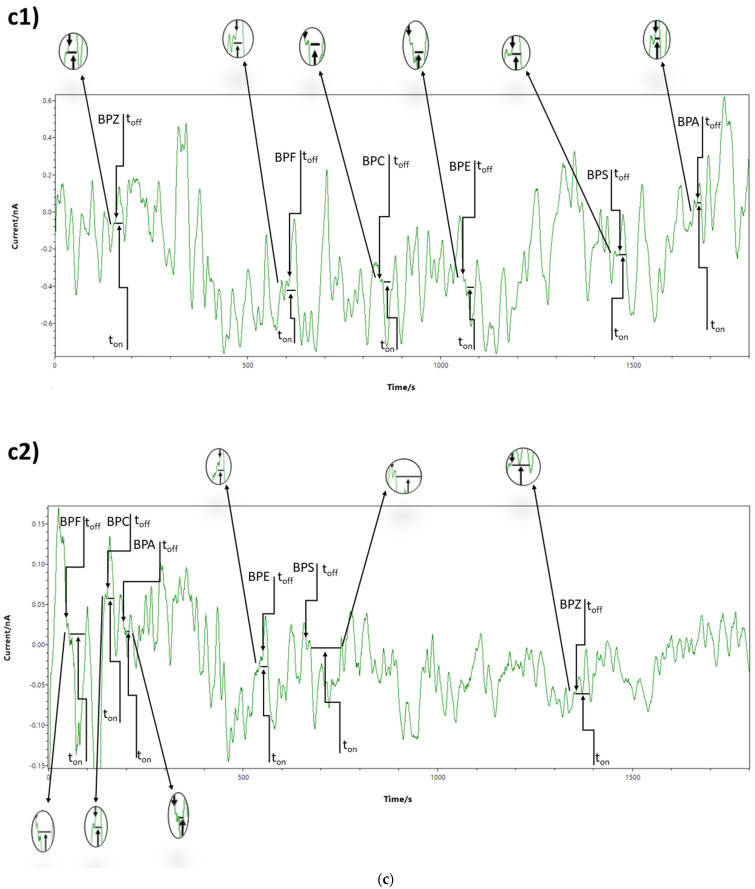

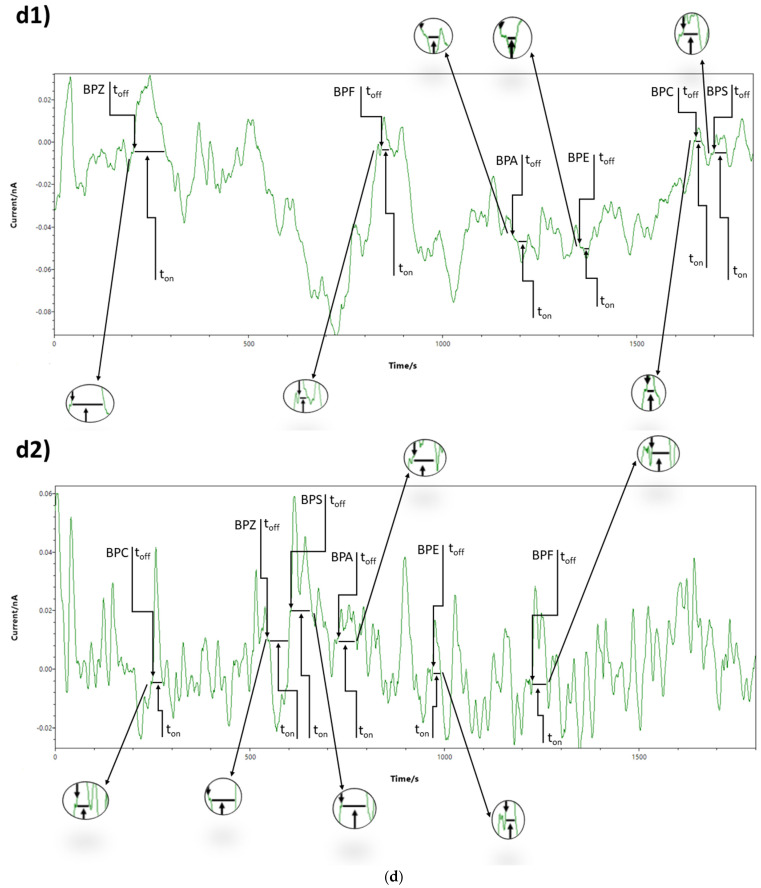

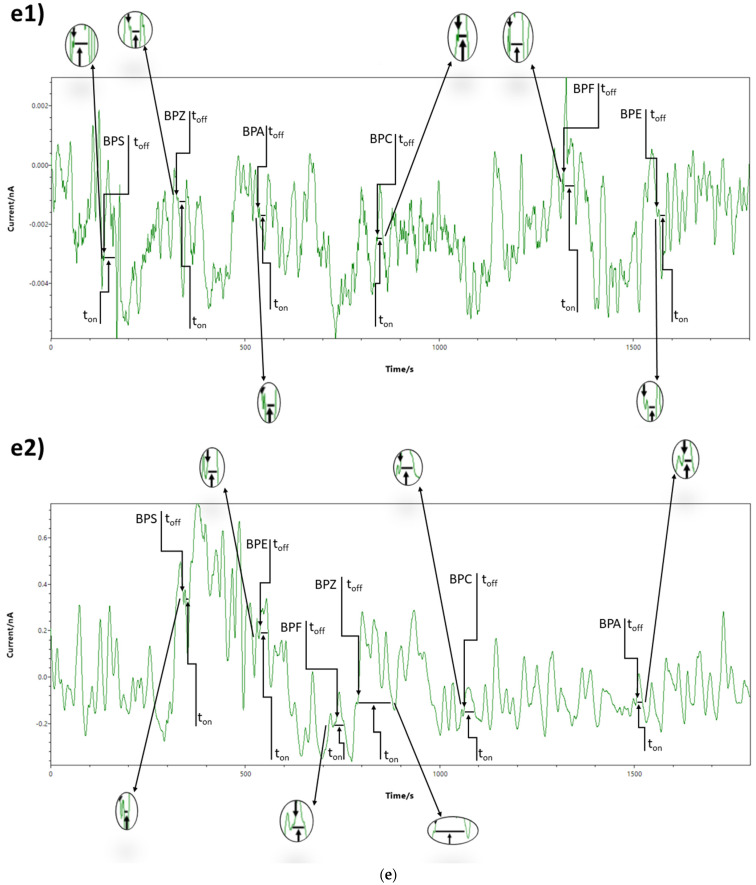

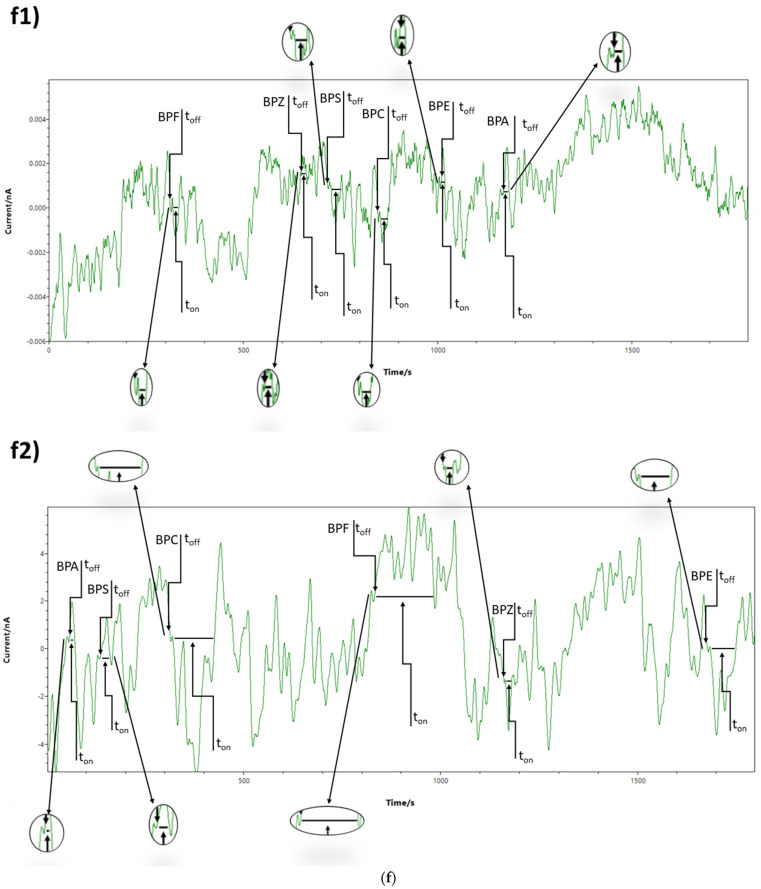
Examples of diagrams obtained for the determination of BPA, BPC, BPE, BPF, BPS, and BPZ in different food samples: (**a**) milk (**a1**)—in a bottle made from polypropylene with a silicon nipple; (**a2**)—in a bottle made from borosilicate glass with a silicon nipple; (**a3**)—in a bottle made from polypropylene with a latex nipple; (**a4**)—in a bottle made from borosilicate glass with a latex nipple; (**a5**)—in a bottle made from polypropylene with a silicon nipple), (**b**) canned pear (**b1**)—fruit; (**b2**)—juice, (**c**) preserved tomatoes (**c1**)—tomato; (**c2**)—juice, (**d**) tuna preserved in its own juice (**d1**)—tuna; (**d2**)—oil, (**e**) tuna preserved in its own juice and oil (**e1**)—tuna; (**e2**)—oil, and (**f**) tuna preserved in oil (**f1**)—tuna; (**f2**)—oil.

All measurements were carried out at 25 °C. A chronoamperometric method was used for the measurements of t_on_ and t_off_ at a constant potential (180 mV vs. Ag/AgCl). A drop of each of the bisphenols in a concentration range of 10^−18^ to 10^−2^ mol L^−1^ was added to the disposable sensor. Equations of calibration for each of the bisphenols were obtained using the linear regression method. The bisphenols were identified in the diagrams based on their t_off_ values (Table 1, Figure 5).

The unknown concentrations of bisphenols were determined from the equations of calibration using the t_on_ values read in the diagram in-between two t_off_ values.

### 2.4. Samples

All samples were purchased from a supermarket. Milk samples were analyzed after the milk was heated to 35 °C in different types of bottles: sample 1 (Figure 5(a1)) in a bottle made from polypropylene with a silicon nipple, sample 2 (Figure 5(a2)) in a bottle made from borosilicate glass with a silicon nipple, sample 3 (Figure 5(a3)) in a bottle made from polypropylene with a latex nipple, and sample 4 (Figure 5(a4)) in a bottle made from borosilicate glass with a latex nipple; all of these bottles were heated in boiling water until the milk reached 35 °C. For sample 5 (Figure 5(a5)), the milk was placed in a bottle made from polypropylene with a silicon nipple and heated to 35 °C using an electrical heating machine designed for baby bottles. Canned pear (Figure 5(b1,b2)) and canned tomatoes (Figure 5(c1,c2)) were analyzed: first the juice, and then the pear and the tomato pieces. Three types of canned tuna fish were analyzed: the first was tuna fish prepared in its own juice (Figure 5(d1,d2)), the second was tuna fish prepared in a mixture of oil and its own juice (Figure 5(e1,e2)), and the third one was preserved only using oil (Figure 5(f1,f2)). For canned tuna fish, the juice as well as the tuna fish were analyzed.

While the milk and the juice from the canned fruits, tomatoes, and the tuna fish were analyzed without any sampling by placing a drop on the 2D disposable sensor, the fruits, tomatoes, and fish were blended and the mixture was placed on the surface of the 2D disposable sensor.

## 3. Results and Discussion

### 3.1. Response Characteristics of the 2D Disposable Stochastic Sensors

Chronoamperometry (performed at a constant potential of 180 mV vs Ag/AgCl) was employed for all measurements. When the potential was applied, the bisphenols went to the electrode interface and penetrated inside the channel—while entering the channel, the intensity of the current becomes zero (the time needed to enter the channel is called the signature of the bisphenol and is marked as t_off_ in the diagrams). While in the channel, the bisphenols undergo binding and redox processes—the time needed to complete the processes is marked on the diagram as t_on_ and is read in-between two t_off_ values.

The response characteristics of the 2D disposable stochastic sensor are presented in Table 1. Differences in the signatures (t_off_ values) proved that the sensor could differentiate between bisphenols A, C, E, F, S, and Z. High sensitivity values and low limits of determination were recorded, making their ultrasensitive assay possible in the samples. The limits of quantification allow for their determination in very small concentrations ranging from 1 fmol L^−1^ for BPC and BPE, to 10 fmol L^−1^ for BPA and BPF, and to 1 pmol L^−1^ for BPZ and BPS. Wide linear concentration ranges were obtained for all bisphenols.

Reproducibility studies were performed as follows: 10 sensors were manufactured following the procedure shown in the section on the sensor design. Each of the sensors was evaluated in the same way, and the sensitivities were determined and compared when immersed in each of the bisphenol solutions. The RSD (%) values recorded for the sensitivities were: 0.10% for BPA, 0.11% for BPC, 0.09% for the BPE, 0.13% for BPF, 0.15% for the BPS, and 0.12% for BPZ. The RSD (%) values recorded for the sensitivities proved the reproducibility of the sensor design.

The stability of each 2D sensor (designed for single utilization) was checked as follows: 10 sensors of each type were stored as described in the section on the sensor design. Every day, a different sensor was taken from the storage space and immersed in the solutions containing each of the bisphenols with different concentrations; the sensitivities of each measurement were retained for comparison after all of the sensors were consumed for 30 days. The results recorded at the end of the period showed the high stability of the electrodes in time because the variation of the sensitivities in time was less than 0.30%.

### 3.2. The Selectivity of the 2D Stochastic Sensors

The selectivity is given by the signatures of the bisphenols and of other substances found in the real samples; a difference in the signature values shows that the proposed sensor is selective. The signatures of the analytes did not depend on the matrix from where the analytes were determined, but depended on the length and the volume of the molecules, and their velocity to move inside the channel. Accordingly, all the analytes from a solution went inside the channel in a certain sequence, ordered by the length and the stereochemistry of the molecules. Different signatures (t_off_ values) were recorded for the six bisphenols (Table 1), proving the selectivity of the method versus these bisphenols. Heavy metals such as Cd, Pb, and Hg were checked as possible interferences. The t_off_ values recorded were: 1.9 s for Hg, 2.4 s for Pb, and 3.0 s for Cd—proving that the proposed 2D sensor is selective versus these metals.

### 3.3. The Determination of Bisphenols A, C, E, F, S, and Z in Food Samples

Bisphenols A, C, E, F, S, and Z were analyzed from the following samples: milk, canned pear, canned tomatoes, and three types of canned tuna fish. For the milk samples, the recipients on which the milk was heated (e.g., the material of the bottle and of the nipple) as well as the heating system were considered (see Samples section). For the canned food, all cans were metallic with the inside wall being painted with a paint that may have contained bisphenols. The determination of the bisphenols was performed for each sample before and after the addition of a mixture containing known amounts of BPA, BPC, BPE, BPF, BPS, and BPZ. To determine the concentrations of the bisphenols in the food samples, the signature of each of the bisphenols was identified in the diagram (Figure 5), after which the t_on_ value was read and introduced in the equation of calibration as described in the stochastic method above-mentioned. The recovered amount of each bisphenol was compared with the amount added, and %, recovery, and %, RSD values were calculated after 10 measurements. The results presented in Table 2 show that the bisphenols could be reliably determined in all of the tested samples of the milk, canned pear, canned tomatoes, and three types of canned tuna fish.

Compared with the results reported earlier for the chromatographic analysis [10,15,18], the spectrophotometric [12], and especially the electrochemical [9,11,13,17], determination of different bisphenols in the environment or different types of food of samples, the proposed method showed a very low limit of determination, especially for BPA, BPC, BPE, and BPF, a far higher sensitivity, and wider limits of determination. Additionally, it is very important to mention that this is the only method of analysis able to perform a reliable simultaneous determination of the six bisphenols: A, C, E, F, S, and Z.

## 4. Conclusions

A portable device based on the utilization of a 2D disposable paper stochastic sensor was proposed for the fast ultrasensitive screening of food samples for bisphenols. This is the first method that was able to reliably and simultaneously analyze six bisphenols: bisphenol A, bisphenol C, bisphenol E, bisphenol F, bisphenol S, and bisphenol Z in milk, canned pear, canned tomatoes, and canned tuna fish. This portable method can be applied for the assay of bisphenols A, C, E, F, S, and Z in the food on the shelves of the supermarkets at any time. Due to its wireless access, the data may be set to be automatically submitted to the customer protection department, which can take action to retract the products if the amounts admitted are exceeded.

## Figures and Tables

**Table 1:** Response characteristics of the 2D disposable sensors used for the assay of BPA, BPC, BPE, BPF, BPS, and BPZ.

BP	Signature, t_off_, s	Equation of Calibration * R	Linear Concentration Range mol L^−1^	Sensitivity s^−1^ mol^−1^ L	Limit of Quantification mol L^−1^
A	1.0	1/t_on_ = 0.15 + 1.52 × 10^11^ C R = 0.9999	10^−14^–10^−9^	1.52 × 10^11^	10^−14^
C	0.5	1/t_on_ = 0.59 + 1.08 × 10^11^ C R = 0.9996	10^−15^–10^−5^	1.08 × 10^11^	10^−15^
E	1.2	1/t_on_ = 0.23 + 1.20 × 10^13^ C R = 0.9992	10^−15^–10^−9^	1.20 × 10^13^	10^−15^
F	1.5	1/t_on_ = 0.03 + 9.72 × 10^11^ C R = 0.9995	10^−14^–10^−12^	9.72 × 10^11^	10^−14^
S	0.8	1/t_on_ = 0.38 + 1.05 × 10^9^ C R = 0.9999	10^−12^–10^−5^	1.05 × 10^9^	10^−12^
Z	0.3	1/t_on_ = 0.33 + 3.34 × 10^9^ C R = 0.9998	10^−12^–10^−7^	3.34 × 10^9^	10^−12^

* t_on_ = s; C = mol L^−1.^

**Table 2:** The determination of bisphenols from different food samples.

Sample	BP	Amount Added (pmol)	Amount Found (pmol)	%, Recovery	%, RSD
			Milk		
1	A	-	1.13	-	-
	C	-	0.93	-	-
	E	-	1.20	-	-
	F	-	1.00	-	-
	S	-	1.38	-	-
	Z	-	1.14	-	-
	A	10.00	9.95	99.50	1.05
	C	0.10	0.098	98.23	1.02
	E	1.00	0.93	93.21	0.98
	F	100.00	99.87	99.87	1.10
	S	100.00	99.50	99.50	1.12
	Z	100.00	99.73	99.73	1.00
2	A	-	1.07	-	-
	C	-	0.97	-	-
	E	-	0.87	-	-
	F	-	1.02	-	-
	S	-	1.12	-	-
	Z	-	1.26	-	-
	A	10.00	9.76	97.61	1.02
	C	0.10	0.097	97.82	1.02
	E	1.00	0.95	95.32	1.07
	F	100.00	99.76	99.76	1.03
	S	100.00	99.37	99.37	0.99
	Z	100.00	99.80	99.80	1.12
3	A	-	1.00	-	-
	C	-	0.55	-	-
	E	-	0.95	-	-
	F	-	1.30	-	-
	S	-	1.19	-	-
	Z	-	1.54	-	-
	A	10.00	9.91	99.12	1.01
	C	0.10	0.093	93.47	1.05
	E	1.00	0.98	98.98	1.07
	F	100.00	99.70	99.70	0.97
	S	100.00	99.35	99.35	1.03
	Z	100.00	99.40	99.40	1.02
4	A	-	2.18	-	-
	C	-	1.08	-	-
	E	-	0.76	-	-
	F	-	1.39	-	-
	S	-	1.99	-	-
	Z	-	1.87	-	-
	A	10.00	9.93	99.31	0.57
	C	0.10	0.095	95.21	1.06
	E	1.00	0.97	97.00	0.93
	F	100.00	99.99	99.99	0.92
	S	100.00	99.95	99.95	1.01
	Z	100.00	99.87	99.87	1.12
5	A	-	1.22	-	-
	C	-	0.59	-	-
	E	-	0.66	-	-
	F	-	2.20	-	-
	S	-	1.08	-	-
	Z	-	1.99	-	-
	A	10.00	9.99	99.93	0.91
	C	0.10	0.095	95.00	1.03
	E	1.00	0.92	92.47	1.05
	F	100.00	99.47	99.47	0.98
	S	100.00	99.80	99.80	1.02
	Z	100.00	99.73	99.73	1.05
Canned pear					
Juice	A	-	4.22	-	-
	C	-	1.15	-	-
	E	-	3.37	-	-
	F	-	2.97	-	-
	S	-	3.07	-	-
	Z	-	2.05	-	-
	A	10.00	9.47	94.71	1.03
	C	0.10	0.092	92.95	1.05
	E	1.00	0.95	95.73	0.95
	F	100.00	99.35	99.35	0.98
	S	100.00	99.74	99.74	1.02
	Z	100.00	99.57	99.57	1.03
Fruit	A	-	4.50	-	-
	C	-	1.97	-	-
	E	-	1.66	-	-
	F	-	2.32	-	-
	S	-	1.98	-	-
	Z	-	1.53	-	-
	A	10.00	9.20	92.01	1.01
	C	0.10	0.091	91.93	1.02
	E	1.00	0.91	91.46	1.01
	F	100.00	92.46	92.46	0.98
	S	100.00	93.50	93.50	0.99
	Z	100.00	92.95	92.95	0.99
Preserved tomatoes					
Juice	A	-	2.22	-	-
	C	-	4.59	-	-
	E	-	3.66	-	-
	F	-	2.20	-	-
	S	-	2.09	-	-
	Z	-	2.00	-	-
	A	10.00	9.37	93.73	1.00
	C	0.10	0.095	95.95	1.07
	E	1.00	0.93	93.02	0.95
	F	100.00	93.47	93.47	0.96
	S	100.00	93.51	93.51	1.03
	Z	100.00	94.02	94.02	1.05
Fruit	A	-	5.32	-	-
	C	-	1.06	-	-
	E	-	3.52	-	-
	F	-	1.51	-	-
	S	-	1.03	-	-
	Z	-	2.57	-	-
	A	10.00	9.23	92.37	1.03
	C	0.10	0.091	91.29	1.02
	E	1.00	0.94	94.05	0.98
	F	100.00	93.92	93.92	0.09
	S	100.00	94.07	94.07	0.91
	Z	100.00	93.05	93.05	0.90
Tuna preserved in its own juice					
Own juice	A	-	3.19	-	-
	C	-	1.35	-	-
	E	-	1.53	-	-
	F	-	1.54	-	-
	S	-	1.22	-	-
	Z	-	2.98	-	-
	A	10.00	9.47	94.72	1.03
	C	0.10	0.095	95.00	1.02
	E	1.00	0.96	96.03	0.99
	F	100.00	95.97	95.97	0.95
	S	100.00	96.03	96.03	0.98
	Z	100.00	96.73	96.73	1.04
Tuna	A	-	5.22	-	-
	C	-	0.21	-	-
	E	-	0.32	-	-
	F	-	2.25	-	-
	S	-	1.19	-	-
	Z	-	2.75	-	-
	A	10.00	9.57	95.72	1.01
	C	0.10	0.093	93.03	1.01
	E	1.00	0.98	98.42	0.98
	F	100.00	96.97	96.97	1.03
	S	100.00	96.95	96.95	1.02
	Z	100.00	98.42	98.42	1.01
Tuna preserved in its own juice and oil					
Own juice and vegetal oil	A	-	1.38	-	-
	C	-	0.23	-	-
	E	-	0.15	-	-
	F	-	1.94	-	-
	S	-	1.28	-	-
	Z	-	1.55	-	-
	A	10.00	9.91	99.14	0.97
	C	0.10	0.091	91.27	0.94
	E	1.00	0.95	95.43	1.03
	F	100.00	99.95	99.95	0.95
	S	100.00	99.87	99.87	0.98
	Z	100.00	99.57	99.57	0.99
Tuna	A	-	2.59	-	-
	C	-	0.25	-	-
	E	-	0.87	-	-
	F	-	2.95	-	-
	S	-	3.06	-	-
	Z	-	4.21	-	-
	A	10.00	9.93	99.37	1.03
	C	0.10	0.092	92.08	1.02
	E	1.00	0.97	97.97	1.00
	F	100.00	99.48	99.48	0.98
	S	100.00	99.57	99.57	0.98
	Z	100.00	99.47	99.47	1.05
Tuna preserved in oil					
Oil	A	-	7.13	-	-
	C	-	0.16	-	-
	E	-	0.21	-	-
	F	-	5.20	-	-
	S	-	1.00	-	-
	Z	-	3.27	-	-
	A	10.00	9.98	99.83	1.01
	C	0.10	0.097	97.97	1.00
	E	1.00	0.98	98.05	0.95
	F	100.00	98.99	98.99	1.03
	S	100.00	99.01	99.01	1.02
	Z	100.00	99.12	99.12	1.05
Tuna	A	-	5.15	-	-
	C	-	4.76	-	-
	E	-	2.90	-	-
	F	-	2.17	-	-
	S	-	9.12	-	-
	Z	-	5.07	-	-
	A	10.00	9.97	99.73	1.00
	C	0.10	0.096	96.95	0.99
	E	1.00	0.98	98.14	0.98
	F	100.00	99.84	99.84	1.03
	S	100.00	99.15	99.15	1.02
	Z	100.00	99.12	99.12	1.05

## Data Availability

Not applicable.
